# Naringin and denosumab ameliorate osteoporosis and suppress the aldosterone-MR/SGK1 signaling axis by modulating the bone-kidney interorgan communication in Orchiectomized rats

**DOI:** 10.3389/fendo.2026.1794408

**Published:** 2026-06-30

**Authors:** Shishuo Xiong, Guoying Wu, Yelin Zhong, Rong Xiang, Yukai Zhang, Haiwei Guo, Zehua Guo, Wenhao Lu, Qing Lan, Yongzhen Chen, Ying Li

**Affiliations:** 1 The Third Clinical Medical College, Guangzhou University of Chinese Medicine, Guangzhou, China; 2 Endoscopy Center, Guangdong Provincial Hospital of Integrated Traditional Chinese and Western Medicine, Foshan, China; 3 Spine Department, The Third Affiliated Hospital of Guangzhou University of Chinese Medicine, Guangzhou, China

**Keywords:** aldosterone, bone-kidney axis, denosumab, kidney governing bones, mineralocorticoid receptor (MR), naringin, osteoporosis

## Abstract

While aldosterone’s detrimental role in osteoporosis through activation of the mineralocorticoid receptor (MR) and its downstream target SGK1 is recognized, it remains unclear whether bone itself can reciprocally regulate systemic aldosterone metabolism. Inspired by the traditional Chinese medicine theory of “Kidney Governing the Bones,” this study investigated the hypothesis of a bidirectional “bone-kidney” axis, examining whether anti-osteoporosis treatments could systemically modulate the aldosterone-MR/SGK1 axis while improving bone mass. Using an orchiectomized (ORX) aging male rat model, we intervened with either the flavonoid naringin (200 mg/kg/d, gavage) or the anti-RANKL antibody denosumab (6.3 mg/kg, s.c.). Micro-CT analysis confirmed that naringin significantly increased bone mineral density (BMD) and improved trabecular microarchitecture, with a similar exploratory trend in the denosumab group (e.g., increased bone volume fraction (BV/TV) and trabecular number (Tb.N), decreased trabecular separation (Tb.Sp)). Notably, ELISA revealed that the ORX-induced elevation in serum aldosterone was markedly suppressed by both naringin and denosumab. At the molecular level, qPCR and Western blot analyses demonstrated that both treatments inhibited the upregulated MR/SGK1 signaling pathway in the kidney and suppressed MR expression in bone tissue, concurrently correcting the imbalance in Receptor Activator of Nuclear Factor Kappa-B Ligand (RANKL)/Runt-related transcription factor 2 (Runx2) expression in femur. This study provides the first experimental evidence that bone- targeted interventions can downregulate systemic aldosterone levels and its key signaling pathway, suggesting a functional bone- kidney crosstalk that warrants further mechanistic investigation.

## Introduction

Osteoporosis is a systemic skeletal disease characterized by reduced bone mass and deterioration of bone microstructure, which significantly increases the risk of fractures and severely impacts patients’ quality of life (1). Beyond classical risk factors such as age and sex hormone levels, the role of the renin-angiotensin-aldosterone system (RAAS) in bone metabolism has garnered increasing attention in recent years (2, 3). Aldosterone, a key component of RAAS, maintains homeostasis of fluid volume and electrolyte metabolism (4)by binding to the mineralocorticoid receptor (MR) to regulate the reabsorption of water, Na+, and K+ (5). Substantial evidence now indicates a significantly higher incidence of osteoporosis in patients with primary aldosteronism (PA) (6, 7). The core mechanism involves two primary pathways: firstly, excess aldosterone activates renal MR, promoting urinary calcium excretion via the serum and glucocorticoid-regulated kinase 1 (SGK1) - epithelial sodium channel (ENaC) pathway (8), leading to hypocalcemia and subsequent secondary hyperparathyroidism, thereby accelerating bone resorption. Secondly, aldosterone can directly act on MR in bone tissue (9, 10), inhibiting osteoblast function. These combined pathways result in bone resorption exceeding formation, ultimately leading to osteoporosis. These studies clearly delineate a unidirectional influence pathway from the ‘kidney/adrenal gland → bone’.

However, a crucial scientific question has long been overlooked: can the health status of bone inversely regulate systemic aldosterone metabolism? The hypothesis of a “bone-kidney” bidirectional communication has recently begun to emerge. A cross-sectional study of 324 subjects found that plasma aldosterone concentration (PAC) and the aldosterone/renin ratio (ARR) increased as bone mineral density (BMD) decreased, with reduced BMD being closely associated with increased PAC (11). Furthermore, a groundbreaking recent study by Hu et al. (12) overturned the traditional perspective. They discovered a negative correlation between PAC and BMD in postmenopausal osteoporotic women, with a strikingly high false-positive rate of 58% for ARR. More notably, following anti-osteoporosis treatment, patients exhibited a significant decrease in PAC alongside improved BMD. This critical finding strongly suggests that bone itself may function as an active endocrine regulatory organ, and that the osteoporotic state can send feedback signals to the kidneys/adrenal glands, downregulating aldosterone levels – implying the existence of a bidirectional regulatory circuit, a “bone-adrenal axis”.

Intriguingly, this holistic view of “bone-kidney” bidirectional interaction finds resonance not only in modern medicine (13), but also aligns remarkably well with the core theory of “the kidney governing the bones” in Traditional Chinese Medicine (TCM) (14). Su Wen · Xuan Ming Wu Qi explicitly states that “the kidney governs the bones” (15), and Su Wen · Liu Jie Zang Xiang Lun further elaborates that “the kidney is the root of storage and sealing, the residence of essence, and its manifestation is in the bones,” indicating that kidney essence is the material basis for strong bones. Conversely, Ling Shu · Ben Shen states, “impairment of essence leads to bone ache, weakness, and cold limbs,” describing not only the pathological transmission where consumption of kidney essence inevitably affects the bones but also implying that bone pathology is a key indicator of kidney qi decline (16); the two are intimately interconnected, physiologically interdependent and pathologically linked (17, 18). Numerous previous studies have also demonstrated the therapeutic and preventive effects of kidney-tonifying Chinese herbs on osteoporosis (19, 20).

Based on these clinical findings and theoretical foundations, we propose the following scientific hypothesis: Effective anti-osteoporosis treatment, while improving bone mass, can inversely reduce serum aldosterone levels by inhibiting the MR/SGK1 signaling pathway within the “bone-kidney” axis. To test this hypothesis, this study utilized an osteoporotic model of aged orchiectomized male rats, intervening with the TCM active monomer Naringin (21, 22) and the anti-RANKL monoclonal antibody Denosumab (23, 24), respectively. It aims to demonstrate that these two anti-osteoporosis drugs, with different mechanisms of action, can both achieve the common effect of reducing aldosterone levels by suppressing the expression of MR in bone tissue and the renal MR/SGK1 signaling axis. This study not only provides key experimental evidence and a molecular mechanism for “bone-kidney” bidirectional communication but also endows the TCM theory of “the kidney governing the bones” with new scientific meaning from the perspective of modern biology. It further suggests the potential value of anti-osteoporosis therapy in preventing aldosterone-related bone metabolic disorders.

## Materials and methods

### Apparatus

The following instruments were used: High-speed refrigerated centrifuge (Baiteke, Wuxi, China, Model CR1260); UV spectrophotometer (Baiteke, Wuxi, China, Model ND5000); Horizontal nucleic acid electrophoresis tank (Dongfang Ruili, Beijing, China, Model DYC-SUB2); Electrophoresis power supply (Dongfang Ruili, Beijing, China, Model DYY-600E); Gel imaging system (JUNYI, Beijing, China, Model JY04S-3C); PCR instrument (LongGene, Hangzhou, China, Model A200); Real-time quantitative PCR system (Fusheng, Changzhou, China, Model FS384); Electronic balance (Youke, Shanghai, China, Model FA2204B); Microplate reader (Juchuang Jiaheng, Qingdao, China, Model JC-1086A); Benchtop high-speed refrigerated centrifuge (BIOBASE, Jinan, China, Model TGL-16E); Benchtop centrifuge (Dragon Lab, Beijing, China, Model DM0424); Water purification system (Shenyuan, Chengdu, China, Model SYS-II); Laboratory ice flaker (Guoyi, Xiamen, China, Model GYXH-50); Mini centrifuge (Dragon Lab, Beijing, China, Model D1008); Vortex mixer (Dragon Lab, Beijing, China, Model MX-F); Magnetic stirrer (Dragon Lab, Beijing, China, Model MS-H-S); Orbital shaker (Dragon Lab, Beijing, China, Model SK-L180-Pro); Electrophoresis power supply (Liuyi, Beijing, China, Model DYY-6C); Dual vertical electrophoresis cell (Liuyi, Beijing, China, Model DYCZ-24DN); Transfer electrophoresis cell (Liuyi, Beijing, China, Model DYCZ-40D); pH meter (Youke, Shanghai, China, Model P901); Image acquisition system (Clinx, Shanghai, China, Model ChemiScope 6100); High-speed tissue homogenizer (Jingxin, Shanghai, China, Model HLK-s-4). Additional equipment included a microplate reader (450 nm), high-precision pipettes and tips (covering ranges 0.5-10 µL, 2-20 µL, 20-200 µL, 200-1000 µL), a 37 °C incubator, and centrifuges.

### Chemicals and reagents

The Aldosterone (ALD) ELISA kit, Procollagen Type I N-terminal Propeptide (PINP) ELISA kit, C-terminal telopeptide of type I collagen (CTX-I) ELISA kit, Tartrate-resistant acid phosphatase 5b (TRACP-5b) ELISA kit, were purchased from Jiangsu Meimian Industrial Co., Ltd. (Jiangsu, China). The blood calcium concentration assay kit, (using biochemical methods) were obtained from Jiangsu Aidi Biotechnology Co., Ltd. (Jiangsu, China).The Tissue/Cell RNA Extraction Kit (Genenode, Beijing, China, Cat# R2104), First Strand cDNA Synthesis Kit with gDNA Eraser (Genenode, Beijing, China, Cat# 4202), and 2× SYBR Green qPCR Mix (Genenode, Beijing, China, Cat# 4302) were employed for RNA and cDNA work. The following reagents were also used: RIPA lysis buffer (Genenode Biotech LTD, Beijing, China, Cat# CBW0011); 50× cocktail protease inhibitor (Genenode Biotech LTD, Beijing, China, Cat# CBW0018); PMSF (100 mM) (Genenode Biotech LTD, Beijing, China, Cat# CBW0017); Phosphatase inhibitor cocktail (Genenode Biotech LTD, Beijing, China, Cat# CBW0019); 5× protein loading buffer (Genenode Biotech LTD, Beijing, China, Cat# CBW0029); SDS-PAGE Gel Preparation Kit (Genenode Biotech LTD, Beijing, China, Cat# CBW0001-CBW0010); Nuclear and Cytoplasmic Protein Extraction Kit (Genenode Biotech LTD, Beijing, China, Cat# CBW0016); BCA protein assay kit (Genenode Biotech LTD, Beijing, China, Cat# CBW0020); Protein molecular weight marker (Thermo Fisher Scientific, Waltham, USA, Cat# 26616/26619); PVDF membrane (0.45 µm) (Millipore, Billerica, USA, Cat# IPVH00010); PVDF membrane (0.22 µm) (Millipore, Billerica, USA, Cat# ISEQ00010); BSA (MERCK, Darmstadt, Germany, Cat# V900933); TWEEN 20 (Solarbio, Beijing, China, Cat# T8220); Supersensitive ECL chemiluminescence substrate (Beyotime, Shanghai, China, Cat# P0018M); β-actin antibody (Proteintech, Wuhan, China, Cat# 81115-1-RR); GAPDH antibody (Proteintech, Wuhan, China, Cat# 60004-1-1g); HRP-conjugated goat anti-rabbit IgG secondary antibody (KPL, Gaithersburg, USA, Cat# 074-1506); Absolute ethanol (Sinopharm Chemical Reagent Co., Ltd., Shanghai, China, Cat# 10009218); Transfer buffer (Genenode Biotech LTD, Beijing, China, Cat# CBW0095); Electrophoresis buffer (Genenode Biotech LTD, Beijing, China, Cat# CBW0040); TBS buffer (Genenode Biotech LTD, Beijing, China, Cat# CBW0089).

### Animal study

Forty 3-month-old specific pathogen-free (SPF) male Sprague-Dawley rats (weighing 180–220 g) were obtained from the Laboratory Animal Center of Guangzhou University of Chinese Medicine. The animals were housed under standard SPF conditions: temperature of 23 ± 3 °C, relative humidity of 55 ± 5%, and a 12-hour light/dark cycle. All rats had ad libitum access to standard rodent diet and drinking water. The experimental protocol was approved by the Experimental Animal Ethics Committee of Guangzhou University of Chinese Medicine (Approval No. 20230225017).

After one week of acclimatization, the rats were randomly assigned to two groups: a sham-operated group (Sham, n=10) and an orchiectomized group (ORX, n=30). Prior to surgery, animals were fasted for 12 hours and anesthetized by intraperitoneal injection of 3% sodium pentobarbital (40 mg/kg). Bilateral orchiectomy was performed in the ORX group through a midline scrotal incision. Briefly, the testes were carefully exteriorized, the spermatic cords were ligated with absorbable suture, and the testes were excised (25, 26). In the sham group, an identical procedure was performed, including mobilization of the testes, but without ligation or excision before returning them to the scrotum. All incisions were closed layer by layer. To prevent postoperative infection, every rat received an intramuscular injection of 80,000 units of penicillin once daily for three consecutive days.

**Figure 1:**
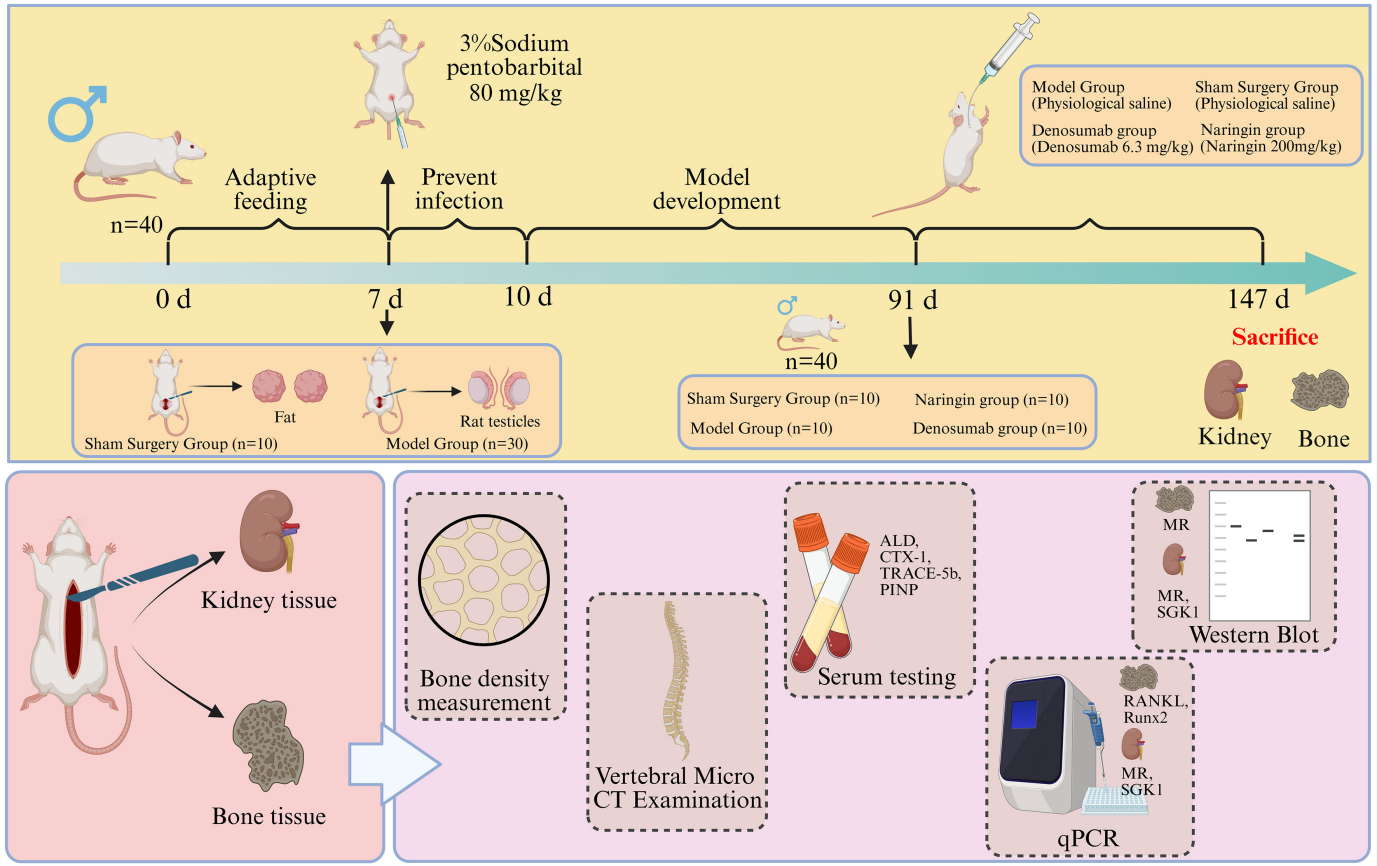
Schematic diagram of the experimental procedure and subsequent analyses following Naringin and Denosumab intervention in male orchiectomized rats.

Twelve weeks after surgery, the ORX rats were further randomized into three treatment subgroups (n=10 per group): the ORX model control group (ORX), the naringin-treated group (ORX + Naringin), and the denosumab-treated group (ORX + Denosumab). Rats in the ORX + Naringin group received naringin (purity ≥ 98%; Solarbio, Beijing, China) via daily oral gavage at a dose of 200 mg/kg for 8 weeks, suspended in 0.5% sodium carboxymethyl cellulose (CMC-Na) solution (27). The Sham and ORX control groups were administered an equal volume of saline by gavage. For the ORX + Denosumab group, a single subcutaneous injection of denosumab (Amgen, USA) was administered at 6.3 mg/kg (dose calculated based on body surface area translation), which was prepared by dissolution in sterile water. Due to the well−known species specificity of denosumab (low cross−reactivity to rat RANKL), results from this group are presented as exploratory only. The corresponding control groups received equivalent volumes of saline subcutaneously. The total duration of all pharmacological interventions was 8 weeks prior to sample collection. The experimental protocol is illustrated in **Figure 1**.

At the end of the 8-week intervention period, all rats were euthanized for terminal sample collection under deep anesthesia. Euthanasia was performed by exsanguination via abdominal aorta puncture after the animals were deeply anesthetized with an intraperitoneal injection of 3% sodium pentobarbital (40 mg/kg), consistent with the anesthetic protocol used for surgery.

### Bone mineral density measurement

After anesthesia, the BMD of rats was measured using dual-energy X-ray absorptiometry (DXA). The data were exported and analyzed using statistical analysis software.

### Vertebral microCT analysis

Following anesthesia and euthanasia, the entire lumbar vertebrae were carefully dissected from the rats and fixed in 4% paraformaldehyde. For MicroCT scanning, the vertebral bodies were placed vertically along their longitudinal axis on the stage of a NEMO Micro CT system (Model NMC-200). The scan was performed under the following conditions: 55 kV source voltage, 145 μA current, and a voxel size of 12.5 μm. A region of interest (ROI) was selected within the trabecular bone of the vertebral body, excluding the cortical areas. Three-dimensional models were then reconstructed to determine key structural parameters, including the bone volume fraction (BV/TV), trabecular number (Tb.N), and trabecular separation (Tb.Sp). The raw data were subsequently reconstructed into three-dimensional images using the accompanying software.

### Serum ELISA assay

Serum concentrations were measured using a commercial ELISA kit according to the manufacturer’s instructions. Briefly, samples were centrifuged at 3000 × g for 10 min, and the supernatant was stored at -20 °C. After bringing all reagents to room temperature, standards or samples (10 μL sample + 40 μL diluent) were added to the pre-coated plate. Then, 100 μL of HRP-conjugated detection antibody was added to each well, and the plate was incubated at 37 °C for 60 min. Following washing, 50 μL each of Substrate A and B were added for a 15-min incubation at 37 °C in the dark. The reaction was stopped with 50 μL stop solution, and the absorbance was measured at 450 nm. A standard curve was plotted to calculate sample concentrations, which were multiplied by the dilution factor (5) for final results.

### Serum calcium assay

Blood samples were centrifuged to obtain serum. The serum calcium concentration was examined using a commercially available assay kit (ADS-W-D039, Microplate Method) according to the manufacturer’s instructions.

### Quantitative real-time PCR

Quantitative real-time PCR (qPCR) was performed using the SYBR Green I method. Briefly, total RNA was extracted from rat bone and kidney tissues using a Tissue & Cell RNA Extraction Kit (Spin-Column, Genenode Biotech LTD, Beijing, China, Cat# R2104). The integrity of the RNA (5 μL per sample) was verified by 1% agarose gel electrophoresis. First-strand cDNA was synthesized from 1 μg of total RNA using the First Strand cDNA Synthesis Kit with gDNA Eraser (Genenode Biotech LTD, Beijing, China, Cat# 4202) in a 20 μL reaction volume under the following conditions: 42 °C for 15–20 min, followed by 85 °C for 5 sec. The synthesized cDNA was diluted 10-fold and used as the template for qPCR amplification. The 10 μL qPCR reaction mixture consisted of 5 μL of 2× SYBR Green qPCR Mix (Genenode Biotech LTD, Beijing, China, Cat# 4302), 0.25 μL each of forward and reverse primers (10 μM), 1 μL of cDNA template, and DEPC-treated ddH_2_O up to 10 μL. The reactions were carried out on an FS384 real-time PCR system (Fusheng, Changzhou, China) using the following protocol: initial denaturation at 95 °C for 3 min; 40 cycles of 95 °C for 15 sec and 60 °C for 30 sec (fluorescence collection). A melt curve analysis was performed post-amplification to confirm the specificity of the PCR products. The relative mRNA expression levels of the target genes (MR, SGK1, RANKL, Runx2) were calculated using the 2−ΔΔCT method, with GAPDH serving as the internal reference control. All primers used in this study are listed in **Table 1**.

**Table 1:** Sequences of primers for RT-qPCR analysis.

Gene	Direction	Sequence(5′-3′)
GAPDH	F	ACGGCAAGTTCAACGGCAC
	R	ACCCCATTTGATGTTAGCGG
MR	F	TGGACAGAGTTGGCAGAGGTT
	R	TCTCGGAAGGTGTAGAAGCAGAAT
SGK1	F	TCGCTCCTGAGGTTCTCCATAAG
	R	GTGCCTTGCTGAGTTGGTGAT
RANKL	F	CATCGGGTTCCCATAAAGTC
	R	TGAAGCAAATGTTGGCGTA
Runx2	F	GCGGACGAGGCAAGAGT
	R	GGAATGCGCCCTAAATCAC

### Western blot analysis

The expression levels of target proteins in rat tissues were determined by Western blot analysis. Briefly, tissue samples were homogenized in ice-cold RIPA lysis buffer (Genenode Biotech LTD, Beijing, China, Cat# CBW0011) supplemented with 1 mM PMSF (Genenode Biotech LTD, Cat# CBW0017) and a protease inhibitor cocktail (Genenode Biotech LTD, Cat# CBW0018). The homogenates were lysed on ice for 30 min and then centrifuged at 12,000 × g for 10 min at 4 °C to collect the supernatant containing the total protein. The protein concentration was quantified using a BCA protein assay kit (Genenode Biotech LTD, Beijing, China, Cat# CBW0020). Subsequently, 30 μg of total protein per sample was denatured by boiling with 5× protein loading buffer (Genenode Biotech LTD, Cat# CBW0029) for 15 min. The proteins were separated by electrophoresis on 10% or 12% SDS-polyacrylamide gels and then transferred onto PVDF membranes (Millipore, USA, 0.45 μm or 0.22 μm) using a wet transfer method. The membranes were blocked with 5% bovine serum albumin (BSA) in TBST for 1 h at room temperature and subsequently incubated with specific primary antibodies (all from Proteintech, Wuhan, China, and diluted at 1:1000) against GAPDH, MR and SGK1, at 4 °C overnight. After washing with TBST, the membranes were incubated with corresponding HRP-conjugated secondary antibodies (KPL, Gaithersburg, USA, diluted at 1:5000) for 1 h at room temperature. Following extensive washes, the protein bands were visualized using a supersensitive ECL chemiluminescence substrate (Beyotime, Shanghai, China, Cat# P0018M) and imaged with a Clinx ChemiScope 6100 chemiluminescence imaging system. The band intensity (gray value) was quantified using AlphaEase FC software, and the relative expression of the target proteins was normalized to that of GAPDH.

### Statistical analysis

All experimental data were collected and analyzed using SPSS Statistics version 27.0 (IBM, USA) and GraphPad Prism version 10.4.1. The data are presented as the mean ± standard deviation (SD). The normality of data distribution was assessed using the Shapiro-Wilk test, and homogeneity of variances was verified using Levene’s test. For multi-group comparisons, one-way analysis of variance (ANOVA) was applied to normally distributed data, with significant differences further analyzed by Tukey’s honest significant difference (HSD) *post-hoc* test. For data that violated the assumptions of normality or homogeneity of variances, the non-parametric Kruskal-Wallis test was used, followed by Dunn’s test with Bonferroni correction for multiple comparisons. A P-value of less than 0.05 was considered statistically significant. .

## Results

### Naringin and denosumab ameliorate bone mineral density and microstructure in orchiectomized rats

**Figure 2:**
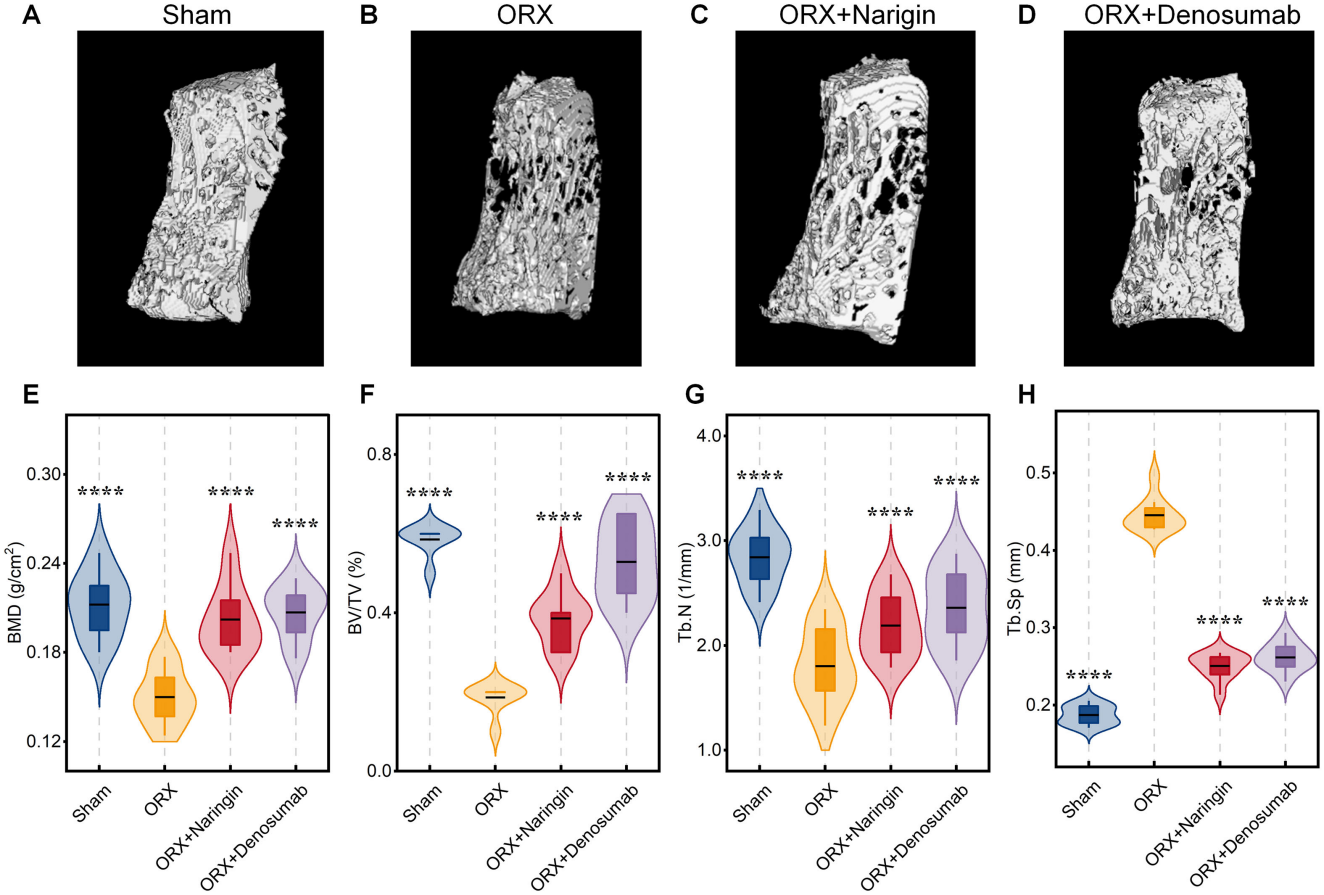
Naringin and denosumab attenuate ORX-induced bone loss and microstructural deterioration. **(A–D)** Representative micro-CT images of rat vertebrae demonstrating the improvement of bone microstructure following Naringin and Denosumab treatment. **(E–H)** Quantitative analysis of bone microstructural parameters: bone mineral density (BMD), bone volume fraction (BV/TV), trabecular number (Tb.N), and trabecular separation (Tb.Sp) (n = 10 per group). Data are expressed as mean ± SD. *p < 0.05, ***p < 0.001, ****p < 0.0001 compared to the ORX group.

To evaluate the interventive effects of Naringin and Denosumab on osteoporosis, the bone microstructure of rat lumbar vertebrae was first analyzed via Micro-CT. Three-dimensional reconstruction of the lumbar vertebral bodies revealed that, compared to the sham group (**Figure 2A**), the model group (**Figure 2B**) exhibited sparse and discontinuous bone networks, thinned trabeculae, a significant reduction in trabecular number, and increased trabecular separation. Combined with the BMD results (**Figure 2E**), these findings confirmed successful model establishment. In contrast, the deterioration of bone microstructure was markedly improved in the Naringin treatment group, and a similar trend was observed in the Denosumab group (**Figures 2C, D**). Given the species limitation of denosumab, the latter finding should be interpreted with caution. Nevertheless, the naringin data consistently support a rescuing effect on bone loss induced in aged orchiectomized male rats. BMD measurements showed that the ORX, ORX+Naringin, and ORX+Denosumab groups all had significantly lower BMD compared to the Sham group, with the most pronounced reduction observed in the model group. Relative to the model group, both treatment groups showed a significant increase in BMD (**Figure 2E**). Quantitative histomorphometric analysis provided statistical support for the qualitative observations (**Figures 2F–H**). The ORX group demonstrated significantly lower BV/TV and Tb.N, and higher Tb.Sp (**Figures 2F–H**). In contrast, both Naringin and Denosumab treatments significantly reversed this bone loss. Animals treated with Naringin exhibited significantly higher BV/TV and Tb.N, and lower Tb.Sp, indicating a notable improvement in bone microstructure following drug intervention (**Figures 2F–H**). Given the exploratory nature of the denosumab arm, this comparison should be interpreted with caution.

### Naringin and denosumab improve bone turnover and reduce serum aldosterone and plasma calcium levels in orchiectomized rats

**Figure 3:**
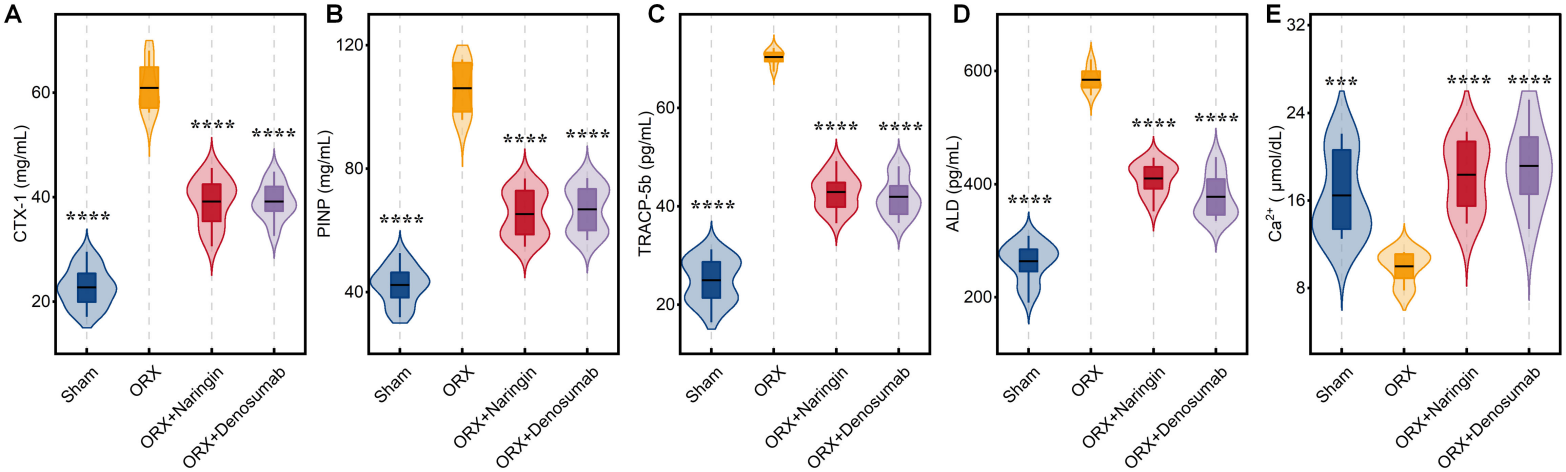
Naringin and denosumab ameliorate the ORX-induced increase in bone turnover markers and aldosterone, and elevate plasma Ca²^+^ levels. **(A–C)** Serum levels of C-terminal telopeptide of type I collagen (CTX-I), tartrate-resistant acid phosphatase 5b (TRACP-5b), and procollagen type I N-terminal propeptide (PINP) in each group (n = 10). **(D)** Serum aldosterone (ALD) levels. **(E)** Plasma Ca²^+^ levels. All values are presented as mean ± SD. *p < 0.05, ***p < 0.001, ****p < 0.0001 vs. the ORX group.

To investigate the regulatory changes of the “bone-kidney” axis under osteoporotic conditions, we measured bone turnover markers (**Figures 3A–C**), serum aldosterone (ALD) concentration (**Figure 3D**), and plasma free calcium levels (**Figure 3E**). Compared to the sham group, the ORX model group showed significantly elevated levels of both bone resorption markers (CTX-I, TRACP-5b; **Figures 3A, C**) and a bone formation marker (PINP; **Figure 3B**), indicating successful induction of a high bone turnover state by orchiectomy, consistent with the aforementioned bone microstructural deterioration. Drug intervention effectively reversed these abnormalities: Naringin treatment significantly suppressed the high bone turnover state. A similar trend was seen with denosumab, but the species limitation of this agent warrants cautious interpretation (**Figures 3A–C**), markedly reducing the levels of CTX-I, TRACP-5b, and PINP compared to the model group.More importantly, alongside the improvement in bone metabolism, both treatments significantly lowered the elevated serum ALD levels (**Figure 3D**) in model rats, accompanied by a recovery in plasma free calcium concentration (**Figure 3E**).We speculate that the trend toward hypocalcemia observed in the ORX group was driven by multiple factors. On one hand, the high bone turnover state led to massive and disordered release of bone calcium, accompanied by renal calcium loss (28). On the other hand, the significantly elevated aldosterone levels in the model group may have activated the renal MR-SGK1 pathway, further promoting urinary calcium excretion (29, 30). The restoration of plasma calcium levels following drug treatment suggests that both Naringin and Denosumab may collectively correct systemic calcium loss by simultaneously inhibiting bone resorption and reducing aldosterone.In summary, our results demonstrate that Naringin and Denosumab, while effectively ameliorating osteoporosis and abnormal bone turnover, can inversely regulate serum aldosterone levels. The concomitant restoration of plasma calcium levels further supports the holistic effect of these two drugs in synchronously correcting disorders of bone metabolism and the aldosterone system via modulation of the “bone-kidney” axis. This finding provides key serological evidence for the “bone-kidney” bidirectional communication hypothesis, indicating that the improvement of skeletal status is sufficient to exert downstream regulatory effects on systemic aldosterone metabolic homeostasis.

### Naringin and denosumab reverse the dysregulation of RANKL and Runx2 gene expression in bone tissue of orchiectomized rats

**Figure 4:**
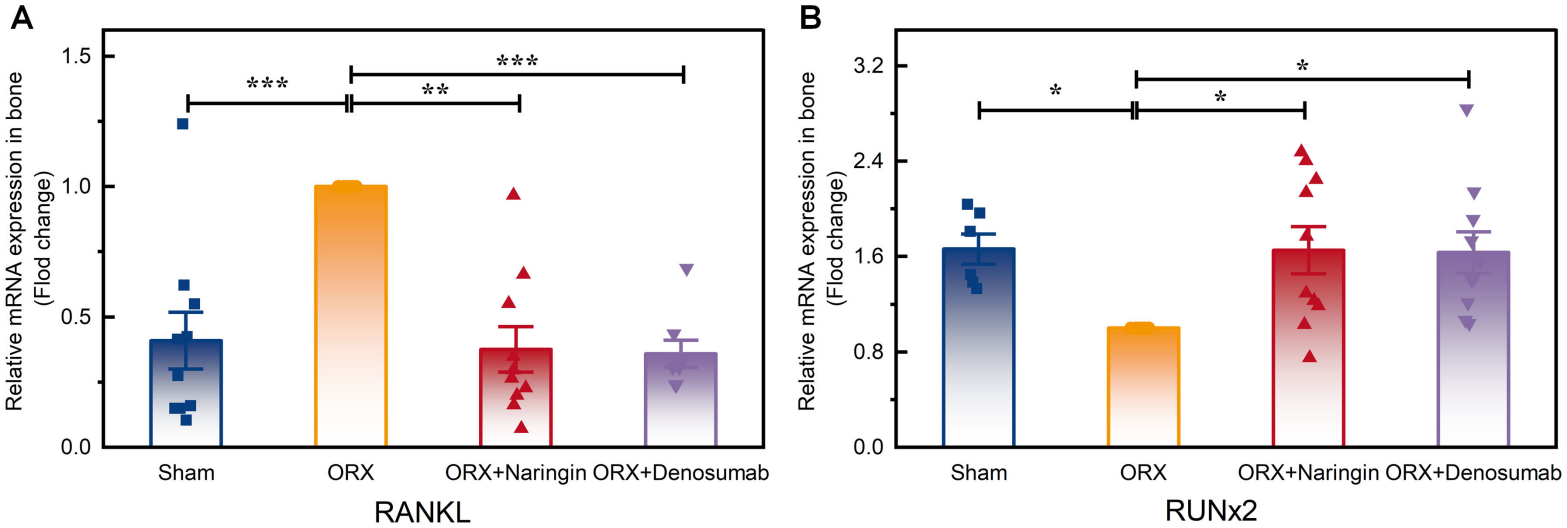
Naringin and denosumab modulate the mRNA expression of RANKL and Runx2 in bone tissue of ORX rats. **(A, B)** mRNA expression levels of **(A)** RANKL and **(B)** Runx2 in bone tissue were analyzed by qPCR. All values are presented as mean ± SD. *p < 0.05, ***p < 0.001, ****p < 0.0001 versus the ORX group.

To elucidate the specific mechanisms underlying the anti-osteoporotic effects of Naringin and Denosumab, we investigated their regulatory roles on the RANKL (**Figure 4A**) and Runx2 (**Figure 4B**) signaling pathways in femoral tissue. At the mRNA level, orchiectomy resulted in a significant increase in RANKL expression (**Figure 4A**) and a significant decrease in Runx2 expression (**Figure 4B**) in the ORX group compared to the Sham group. This expression pattern indicates a disrupted bone metabolic balance under osteoporotic conditions, characterized by the dual pathological features of enhanced osteoclast activity and suppressed osteoblast function. Drug intervention effectively reversed these aberrant gene expressions. Compared to the model group, both Naringin and Denosumab treatments significantly downregulated RANKL mRNA expression and concurrently upregulated Runx2 expression levels (**Figure 4**). These results suggest that both agents may partially correct the imbalance of these key transcriptional regulators, consistent with improved bone metabolism.

### Naringin and denosumab suppress the activation of the MR/SGK1 signaling pathway in the kidney tissue of orchiectomized rats

**Figure 5:**
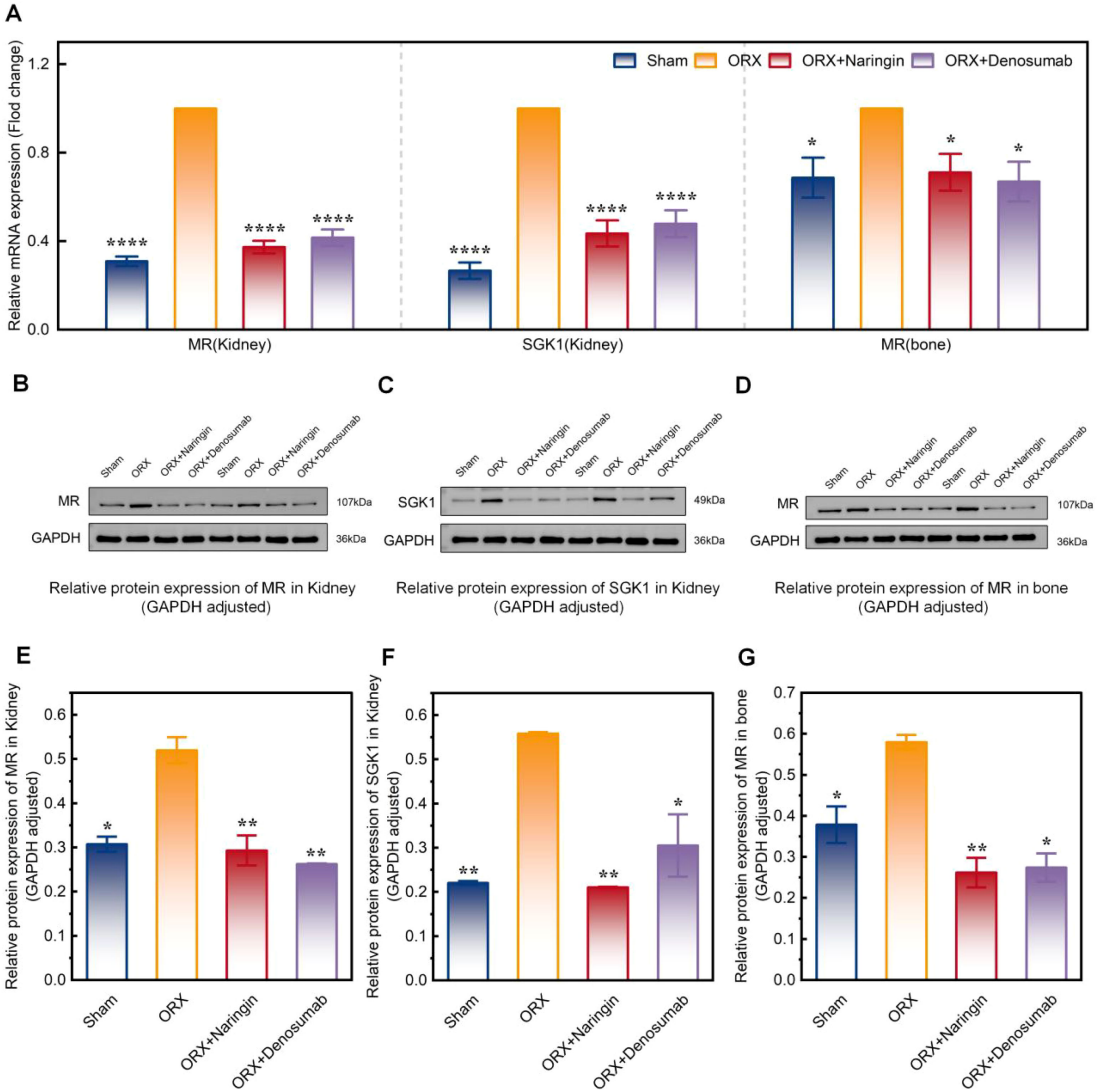
Naringin and denosumab inhibit the renal MR/SGK1 axis and MR expression in bone tissue of ORX rats. **(A)** mRNA levels of MR and SGK1 in kidney and bone tissues were analyzed by qPCR. **(B–D)** Representative Western blots of MR and SGK1 proteins in the indicated tissues. **(E–G)** Quantitative analysis of **(E)** renal MR, **(F)** renal SGK1, and **(G)** bone MR protein levels, normalized to GAPDH. All values are presented as mean ± SD. *p < 0.05, ***p < 0.001, ****p < 0.0001 versus the ORX group.

To investigate the molecular regulatory mechanisms of the “bone-kidney” axis, we further examined the expression of the mineralocorticoid receptor (MR) and its downstream signaling molecule SGK1 in renal tissue using qPCR (**Figure 5A**) and Western blot (**Figures 5B–G**). The results demonstrated that compared to the Sham group, the ORX group exhibited significantly increased expression of both MR and SGK1 in kidney tissue at both the mRNA (**Figure 5A**) and protein levels (**Figures 5B, C, E, F**). MR and SGK1 are recognized as key aldosterone-responsive factors regulating salt-sensitive hypertension and sodium homeostasis (31). This finding aligns with the elevated serum ALD levels observed in the ORX group mentioned above, suggesting that the osteoporotic state is associated with abnormal activation of the renal MR/SGK1 signaling pathway. Following intervention with Denosumab and Naringin, both the mRNA (**Figure 5A**) and protein levels (**Figures 5B, C, E, F**) of MR and SGK1 were markedly reduced. This result indicates that, alongside improving bone metabolism, both anti-osteoporotic agents can inhibit the overactivation of the renal MR/SGK1 signaling axis. These findings provide tissue and molecular evidence that Naringin and Denosumab can modulate the renal MR/SGK1 pathway and intervene in aldosterone system signaling under osteoporotic conditions, offering new experimental support for the “bone-kidney” bidirectional regulatory mechanism.

### Naringin and denosumab inhibit mineralocorticoid receptor expression in the bone tissue of orchiectomized rats

To explore the response of bone tissue itself to aldosterone signaling in osteoporosis, we detected the expression of the mineralocorticoid receptor (MR) in femoral tissue (**Figures 5A, D, G**). Both MR and the glucocorticoid receptor are known to collectively influence bone formation and resorption (32). qPCR and Western blot analyses revealed that compared to the Sham group, the ORX group showed significantly elevated MR expression in bone tissue at both the mRNA (**Figure 5A**, bone MR) and protein levels (**Figures 5D, G**). This result confirms that local MR signaling is also activated in bone tissue under osteoporotic conditions. Drug intervention significantly inhibited this activation. Treatment with either Denosumab or Naringin substantially reduced both mRNA (**Figure 5A**) and protein expression levels (**Figures 5D, G**) of MR in bone tissue compared to the model group. This discovery indicates that successful anti-osteoporosis therapy can not only ameliorate the renal MR/SGK1 pathway but also directly suppress MR expression in bone tissue itself. The ability of Naringin and Denosumab to co-regulate MR and related signaling pathways in both skeletal and renal tissues provides key evidence for elucidating the complete regulatory circuit of “bone-kidney” bidirectional communication.

## Discussion

Our findings first confirm the detrimental role of aldosterone in bone, as evidenced by the co-activation of the MR/SGK1 pathway in kidney and bone tissues alongside bone loss in ORX rats. In the osteoporotic model of orchiectomized male rats, bone loss was accompanied by significantly elevated serum aldosterone levels and concurrent activation of the MR/SGK1 signaling pathway in both bone and kidney tissues. This phenomenon aligns with established pathological mechanisms: in the kidney, aldosterone promotes urinary calcium excretion by activating the MR/SGK1 signal (33); in bone, MR activation directly inhibits osteoblast function (9, 10). These mechanisms are consistent with our observations of downregulated expression of the bone formation marker Runx2, upregulated expression of the bone resorption marker RANKL, and the deterioration of bone microstructure, collectively recapitulating the classical pathological axis of “elevated aldosterone → osteoporosis”. However, the groundbreaking finding of this study lies in revealing the reverse regulatory role of bone on the aldosterone system. Both Naringin and Denosumab directly target bone tissue, and neither acts directly on adrenal aldosterone synthesis. Our experimental results demonstrate that after successfully reversing the osteoporotic phenotype, both drugs concurrently triggered a decrease in serum aldosterone levels, suppressed the renal MR/SGK1 signaling pathway, and inhibited MR expression in bone tissue. This “bone- to- kidney” effect pattern is consistent with a feedback loop, suggesting that bone may not be merely an endpoint for aldosterone action but also a starting point for regulating its systemic homeostasis. This series of findings collectively points to a core conclusion that transcends traditional understanding: bone likely functions as an active endocrine organ that proactively participates in and inversely regulates systemic aldosterone metabolism and the activity of its signaling pathway via a “bone-kidney” axis bidirectional communication circuit. Based on the above evidence, we propose an integrated bidirectional “bone-kidney” axis communication model to elucidate the underlying mechanism. We postulate that under pathological conditions, the deteriorating bone microenvironment sends aberrant signals systemically, potentially through releasing “sick bone factors” or inducing calcium-phosphate metabolic disturbances, promoting aldosterone synthesis and secretion (34, 35). Effective anti-osteoporosis treatment transforms the “sick bone” into a “healthy bone”. This transformation may exert downstream effects through two parallel pathways: Firstly, healthy bone might secrete hypothetical factors that remotely influence aldosterone levels, possibly by acting on the adrenal glands, the kidneys, or indirectly via the calcium−PTH axis (36, 37). The exact mechanism remains unknown. And acting on target organs to reduce the basal activity and responsiveness of their MR/SGK1 pathway. Secondly, the improved bone status directly corrects systemic mineral metabolic disturbances (e.g., blood calcium), thereby eliminating abnormal stimulation of the parathyroid glands (38) and excessive urinary calcium loss (A (39), which indirectly removes aberrant driving forces on the RAAS system (40, 41). These two pathways work synergistically to achieve multi-level, bidirectional inhibition of circulating aldosterone levels and its signaling pathway. Viewed from the perspective of theoretical origins, the findings of this study provide compelling modern biological meaning to the classic TCM theory of “the kidney governing the bones” (18). TCM theory astutely posits the physiological interdependence and pathological interplay between the “kidney” and “bone”. Our experimental results visually reveal, from a modern medical standpoint, that strengthening the physical organ “bone” through pharmacological agents (such as Naringin derived from Chinese herbs) can indeed inversely regulate the aldosterone-MR system, which is closely related to the function of the “kidney”. This not only corroborates the pathological transmission pattern of “bone disease affecting the kidney” but also scientifically validates the feasibility of “treating bone to regulate the kidney” at the therapeutic level, providing an innovative theoretical foundation and experimental basis for the integrated Chinese and Western medicine prevention and treatment of metabolic bone diseases and their systemic comorbidities. The highly consistent changes observed across multiple biological levels—histomorphometry, serology, gene transcription, and protein expression—provide a solid, coherent, and self-consistent chain of evidence for the proposed “bone-kidney” axis model. In summary, the value of this study lies in transcending the linear perspective of “aldosterone unidirectionally acting on bone” and supporting the existence of a functional bone-kidney crosstalk. Our results clearly suggest that skeletal health is an indispensable component in maintaining systemic aldosterone metabolic homeostasis. Consequently, anti-osteoporosis therapy should no longer be viewed merely as a local symptomatic strategy for improving bone mass. It may also represent a potential pathway for systematically regulating aldosterone-related metabolic disorders by remodeling the endocrine function of bone. This shift in conceptual paradigm opens promising new directions for understanding and treating osteoporosis and its comorbidities from a holistic and systemic perspective.

## Limitations of the study

Several limitations of this study should be acknowledged. First, while our interventional design demonstrates a functional relationship between bone status and aldosterone levels, direct mechanistic proof of bone−derived factors controlling adrenal or renal aldosterone signaling is not provided. Second, the use of denosumab in rats is limited by species specificity; therefore, this arm is exploratory and the main conclusions rely on naringin. Third, our bone molecular analysis was restricted to RANKL and Runx2 mRNA; a full panel of osteogenic and osteoclastogenic markers as well as histomorphometry were not performed. Fourth, we did not measure plasma renin activity, angiotensin II, or adrenal CYP11B2 expression, leaving the upstream mechanism of aldosterone reduction unclear. Fifth, our sample size was modest, and we did not monitor dynamic urinary electrolyte excretion to precisely reflect volume status. These limitations should be addressed in future studies.

## Synthesis and proposed model of the bone-kidney bidirectional axis

**Figure 6:**
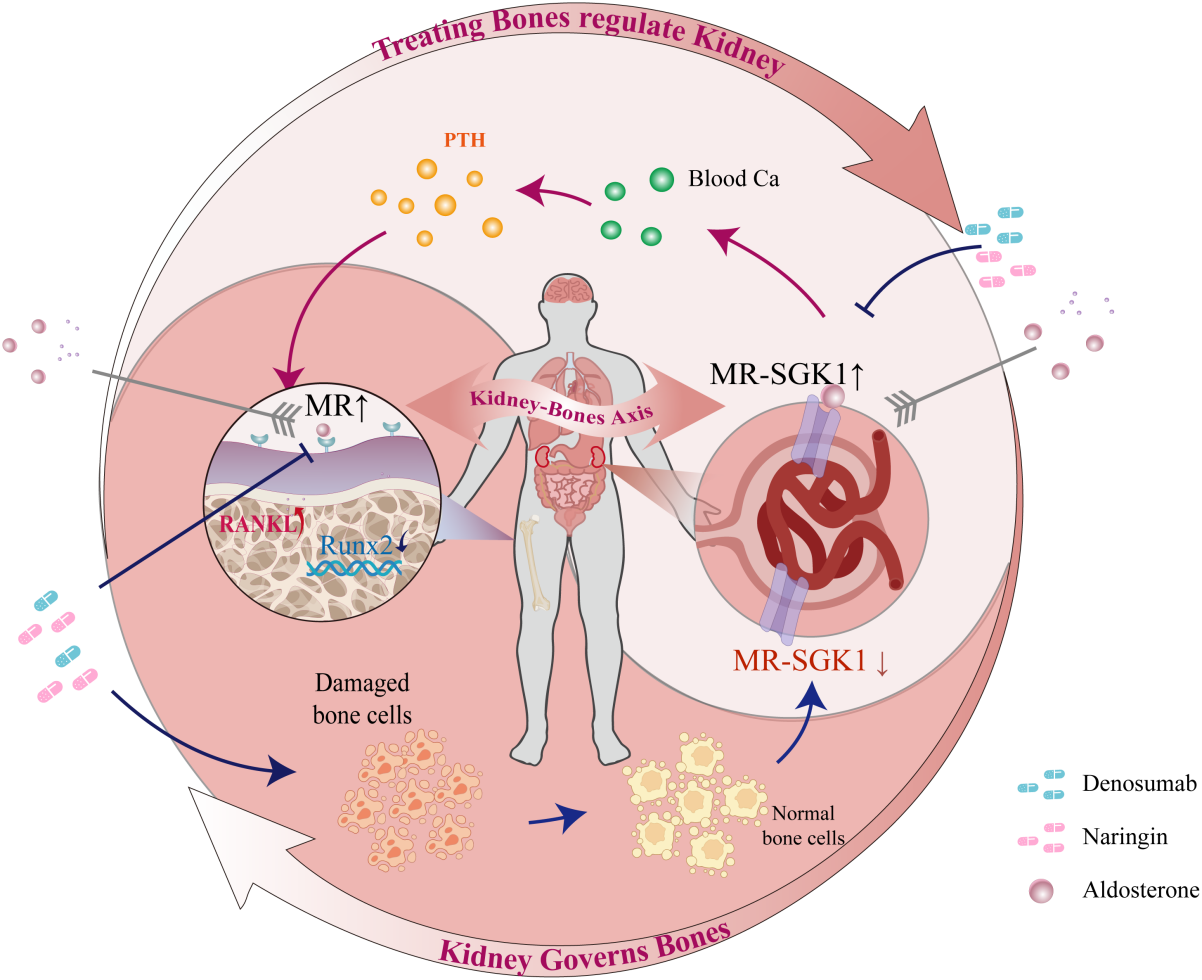
Proposed model of bone–kidney crosstalk. Based on our correlative and interventional findings, the diagram illustrates a hypothesized feedback loop in which “sick bone” may contribute to systemic aldosterone elevation. Direct causality and molecular mediators are not yet established.

In summary, our findings culminate in a holistic “Bone-Kidney Axis” model that integrates both classical and novel regulatory pathways, as illustrated in **Figure 6**. The established unidirectional pathway (Kidney/Adrenal Gland → Bone) is recapitulated in our model: aldosterone, via the mineralocorticoid receptor (MR)/serum- and glucocorticoid-regulated kinase 1 (SGK1) signaling cascade, promotes renal calcium excretion and directly exerts detrimental effects on bone tissue. The groundbreaking insight from our work is the proposal of a reverse regulatory pathway (Bone → Kidney/Adrenal System). We demonstrate that the osteoporotic state of “sick bone” acts as a central trigger, which in turn stimulates systemic aldosterone overproduction and primes the MR/SGK1 pathway in renal tissues while concurrently upregulating MR expression in bone itself. This may contribute to a vicious cycle, reinforcing the pathological signaling in both organs. Critically, our intervention experiments suggest that ameliorating osteoporosis—thereby converting “sick bone” to “healthy bone” with Naringin or Denosumab—effectively suppresses serum aldosterone and concurrently downregulates the MR/SGK1 axis in the kidney and the elevated MR expression in bone. This coordinated correction across tissues is consistent with a functional bidirectional loop, although direct molecular evidence remains to be established, and provides a modern biological basis for the TCM theory while “The Kidney Governs the Bones” explains the classical pathological direction, our findings substantiate the therapeutic principle of “Treating Bone to Regulate the Kidney,” completing the dialogue of the “Bone- Kidney Axis.”

## Conclusion

In conclusion, our study provides experimental evidence consistent with a functional bone–kidney crosstalk that extends the conventional unidirectional view of the aldosterone–bone relationship. Our findings suggest that in an osteoporotic state, a pathologically correlated “bone–kidney” interplay may exist, characterized by elevated serum aldosterone and concomitant upregulation of the MR/SGK1 signaling pathway in both renal and skeletal tissues. Crucially, our findings suggest that this axis is bidirectional. Bone- targeted intervention with naringin (and, in an exploratory manner, with denosumab) while effectively restoring bone mass and microarchitecture, systemically downregulated aldosterone levels and suppressed the MR/SGK1 pathway in the kidneys, and reduced MR overexpression in bone. This work substantiates the novel concept that bone is not merely a passive target but an active regulator in systemic mineralocorticoid homeostasis. The ability of anti-osteoporosis therapy to exert remote effects on the aldosterone system suggests that improving skeletal health is integral to managing broader metabolic disturbances. This paradigm offers a modern biological interpretation of the TCM theory of “Kidney Governing the Bones,” illustrating a potential pathophysiological and therapeutic interconnection between the two organs. While the precise mediators of this interorgan communication remain to be fully elucidated, our data lay the groundwork for future investigations. We hypothesize that the transition from a “sick bone” to a “healthy bone” phenotype may involve the secretion of specific osteogenic factors that modulate remote organ function, but direct evidence for such factors is lacking in the present study. Therefore, identifying these potential “bone-derived factors” through, for instance, unbiased proteomic screening of bone-conditioned serum, represents a critical next step to unravel the complete molecular dialogue within the bone-kidney axis. Ultimately, our findings posit anti-osteoporosis therapy as a potential dual-purpose strategy, not only for increasing bone mass but also for systemically rebalancing aldosterone metabolism, opening new avenues for the holistic treatment of osteoporosis and its comorbidities.

## Data Availability

The datasets presented in this study can be found in online repositories. The names of the repository/repositories and accession number(s) can be found in the article/supplementary material.
